# Investigation of the Impact of Increased Dietary Insoluble Fiber through the Feeding of Distillers Dried Grains with Solubles (DDGS) on the Incidence and Severity of *Brachyspira*-Associated Colitis in Pigs

**DOI:** 10.1371/journal.pone.0114741

**Published:** 2014-12-08

**Authors:** Bailey L. Wilberts, Paulo H. Arruda, Joann M. Kinyon, Tim S. Frana, Chong Wang, Drew R. Magstadt, Darin M. Madson, John F. Patience, Eric R. Burrough

**Affiliations:** 1 Department of Veterinary Pathology, College of Veterinary Medicine, Iowa State University, 1600 S. 16^th^ St, Ames, Iowa, 50011, United States of America,; 2 Department of Veterinary Diagnostic and Production Animal Medicine, College of Veterinary Medicine, Iowa State University, 1600 S. 16^th^ St, Ames, Iowa, 50011, United States of America; 3 Department of Animal Science, Iowa State University, 201H Kildee, Ames, Iowa, 50011, United States of America; National Institute of Agronomic Research, France

## Abstract

Diet has been implicated as a major factor impacting clinical disease expression of swine dysentery and *Brachyspira hyodysenteriae* colonization. However, the impact of diet on novel pathogenic strongly beta-hemolytic *Brachyspira* spp. including “*B*. *hampsonii*” has yet to be investigated. In recent years, distillers dried grains with solubles (DDGS), a source of insoluble dietary fiber, has been increasingly included in diets of swine. A randomized complete block experiment was used to examine the effect of increased dietary fiber through the feeding of DDGS on the incidence of *Brachyspira*-associated colitis in pigs. One hundred 4-week-old pigs were divided into five groups based upon inocula (negative control, *Brachyspira intermedia*, *Brachyspira pilosicoli*, *B. hyodysenteriae* or “*B. hampsonii*”) and fed one of two diets containing no (diet 1) or 30% (diet 2) DDGS. The average days to first positive culture and days post inoculation to the onset of clinical dysentery in the *B. hyodysenteriae* groups was significantly shorter for diet 2 when compared to diet 1 (*P* = 0.04 and *P* = 0.0009, respectively). A similar difference in the average days to first positive culture and days post inoculation to the onset of clinical dysentery was found when comparing the “*B*. *hampsonii*” groups. In this study, pigs receiving 30% DDGS shed on average one day prior to and developed swine dysentery nearly twice as fast as pigs receiving 0% DDGS. Accordingly, these data suggest a reduction in insoluble fiber through reducing or eliminating DDGS in swine rations should be considered an integral part of any effective disease elimination strategy for swine dysentery.

## Introduction

*Brachyspira* spp. are a diverse group of Gram-negative, oxygen-tolerant, anaerobic spirochetes. Included in this genus is *Brachyspira hyodysenteriae*, a causative agent of swine dysentery (SD), which is an economically significant disease of grow-finish swine characterized by severe diarrhea with blood and mucus [1]. *Brachyspira pilosicoli* is the only other *Brachyspira* spp. that has traditionally been recognized as a significant swine pathogen and is the causative agent of porcine colonic spirochetosis (PCS), a condition typically associated with mild colitis and reduced feed efficiency and rate of gain [2].

By the mid-1990s, clinical SD had essentially disappeared from North American swine herds as a result of effective treatment, control, and elimination methods. However, outbreaks of bloody diarrhea have been reported in the United States and Canada since 2007 and these outbreaks have often been associated with “*Brachyspira hampsonii*” infection [3]. Like *B. hyodysenteriae, “B. hampsonii”* is strongly beta-hemolytic on blood agar but can be differentiated by its lack of indole cleavage or by *nox* gene sequencing [3]. Experimental infection with “*B. hampsonii*” strains has consistently resulted in disease similar to, if not indistinguishable from, *B. hyodysenteriae* infection [4]–[6]. Clinical SD associated with infection with either *B. hyodysenteriae* or “*B. hampsonii*” is characterized by gross lesions limited to the large intestine that commonly include variable mucosal thickening, hemorrhage, fibrinonecrotic exudate, and abundant mucus. Microscopically, crypt lumens are often distended with mucus, neutrophils are present in the lamina propria, and silver staining highlights spirochetes within crypt lumens and mucus-producing goblet cells.

Coincident with the recent reemergence of SD has been a change in swine feeding practices to include feeding of ethanol byproducts, such as distillers dried grains with solubles (DDGS), which are frequently added to varying degrees in swine rations. Relatively little is published about the pathogenesis of SD associated with infection with either *B. hyodysenteriae* or “*B. hampsonii*”; however, diet is considered to play a major role in disease expression [7]. The effects of DDGS and other forms of insoluble fiber on the intestinal environment of pigs and other mammals have revealed possible mechanisms by which DDGS may enhance disease expression through alteration of the intestinal environment providing pathogenic *Brachyspira* spp. an optimal niche in which to cause clinical colitis [8].

To the authors' knowledge, the impact of increased insoluble dietary fiber resulting from the inclusion of DDGS in swine diets on the development of *Brachyspira-*associated colitis in swine has not described. Accordingly, this study was undertaken to determine whether feeding DDGS enhances infection and disease development in pigs experimentally infected with *Brachyspira* spp.

## Materials and Methods

### Animals

All procedures were approved by the Institutional Animal Care and Use Committee of Iowa State University (Log Number: 1-12-7283). One hundred crossbred pigs 4 weeks-of-age were obtained from a commercial source with no known history of *Brachyspira-*associated disease. Upon arrival, all pigs received a starter/transition diet and were acclimated to the facility for 2 days. Pigs were then weighed and sorted into ten pens of 10 pigs each to ensure even weight distribution across all pens. Each pen was assigned to group based upon the inoculum (sham, *B. intermedia*, *B. pilosicoli*, “*B. hampsonii*”, or *B. hyodysenteriae*) and diet (diet 1: 0% or diet 2: 30% DDGS) they were to receive. Pigs receiving different inocula were separated by room. Pigs were acclimated to their respective diets for two weeks prior to inoculation and remained on these diets until study termination.

### Diet

Diets were formulated to meet or exceed the nutrient requirements for pigs of this age [9] and prepared in mash form at the Swine Nutrition Farm at Iowa State University (Table 1). Corn DDGS was included in the second diet as an additional source of insoluble fiber. Urriola et al. [10] reported that about 96.6% of total dietary fiber in corn DDGS is insoluble. This add rate would be considered on the high side for pigs of this age, but would not be considered high in commercial practice for early grower diets. Both diets were formulated to contain the same concentration of metabolisable energy (3.4 Mcal/kg), the same level of standardized ileal digestible (SID) lysine (1.23%), the same minimum ratios of other essential amino acids to lysine, all expressed on an SID basis, and the same levels of calcium and available phosphorus. Neutral detergent fiber increased from 7.9% to 14.3% from diet 1 to diet 2; the majority of this increased fiber would be insoluble.

**Table 1:** Ingredient and nutrient composition of experimental diets.

Component	Diet 1 (%)	Diet 2 (%)
**Ingredients**		
Corn, yellow dent	61.13	34.55
Corn DDGS	0	30.00
Soybean meal	20.00	17.50
Fish meal, Menhaden Select	5.66	5.06
Whey, dried	10.00	10.00
l-Lysine HCl	0.31	0.31
dl-Methionine	0.12	0.02
l-Threonine	0.12	0.03
l-Tryptophan	0.02	0.01
Monocalcium phosphate	0.48	0.10
Limestone	0.43	0.70
Salt	0.35	0.35
Vitamin premix^a^	0.23	0.23
Trace mineral premix^b^	0.15	0.15
Soybean oil	1.00	1.00
ME, Mcal/kg	1.54	1.54
Crude Protein, %	19.6	23.9
SID Lysine, %	1.23	1.23
SID Threonine, %	0.76	0.76
SID TSAA, %	0.71	0.71
SID Tryptophan, %	0.21	0.21
ADF, %^c^	3.0	5.7
NDF, %^d^	7.9	14.3
Crude Fat, %	4.6	6.4
Calcium, %	0.70	0.71
Phosphorus, %	0.65	0.67
Sodium, %	0.27	0.33
Chloride, %	0.42	0.46

a Provided per kg of diet: 7,000 IU vitamin A, 800 IU vitamin D3, 57 IU vitamin E, 3.4, menadione, 13 mg riboflavin, 64 mg niacin, 31 mg pantothenic acid, and 57 µg vitamin B_12_.

b Provided per kg of diet: 165 mg Zn as ZnSO_4_, 165 mg Fe as FeSO_4_, 39 mg Mn as MnSO_4_, 17 mg Cu as CuSO_4_, 0.3 mg I as Ca(IO_3_)_2_ and 0.3 mg Se as Na_2_SeO_3_.

c ADF  =  Acid detergent fiber.

d NDF  =  Neutral detergent fiber.

### Inoculum

Isolates used in this study were obtained from the culture collection at the Iowa State University Veterinary Diagnostic Laboratory (ISU VDL). Media, isolates, and challenge inocula were prepared as previously described [4]. The “*B. hampsonii*” clade II isolate (EB107) was recovered from a clinical case of SD in 2011 and was 11^th^ and 12^th^ passage at the time of inoculation. The *B. hyodysenteriae* (B204) isolate was originally recovered from a clinical case of SD in 1972 and was 8 – 10^th^ passage at the time of inoculation. The *B. intermedia* isolate (BR2000) and *B. pilosicoli* isolate (BR2001) originated from pigs with clinical diarrhea and nonsuppurative catarrhal colitis and were 11^th^ and 12^th^ passage, respectively.

### *Brachyspira* spp. isolation and growth

For isolation of *Brachyspira* spp., individual rectal swabs were collected daily starting at 5 days post-inoculation (DPI) and at necropsy. Swabs were plated onto selective agars within 6 hours of collection. Specifically, swabs were plated onto CVS selective agar containing colistin, vancomycin, and spectinomycin; and BJ selective agar containing pig feces extract, spiramycin, rifampin, vancomycin, colistin, and spectinomycin. Plate media used in this study were prepared in-house and passed the quality assurance standards of the ISU VDL. An anaerobic environment was provided by a commercial system (BD GasPak EZ Anaerobe Container System, BD Diagnostic Systems, Sparks, MD) and plates were incubated at 41±1°C. Agar plates were observed for growth at 2, 4, and 6 DPI and, for positive cultures, the degree of beta-hemolysis and the presence or absence of ring phenomenon were recorded. The sample was reported as positive if either selective agar had growth of *Brachyspira* spp. The final positive culture for each pig was speciated using matrix-assisted laser desorption ionization time-of-flight mass spectrometry (MALDI-TOF) as previously described [11]. At necropsy, rectal swabs were also directly plated onto MacConkey's agar and brilliant green agar with novobiocin as well as tetrathionate broth enrichment subcultured to brilliant green agar with novobiocin and XLT4 to test for the presence of *Salmonella* spp.

### Animal inoculation

Pigs were inoculated with an agar slurry as previously described [6]. Briefly, pigs received three doses of inoculum (100 ml/dose) administered via gavage 24 hours apart with each administration preceded by a 12–18 hour fast. A 5-g sample of each inoculum was reserved from which 1 gram was vortexed for 45 seconds in tubes with 9 ml of sterile PBS and a few glass beads. A standard plate count procedure was performed by titration of 1 ml of the vortexed sample into 9 ml and carried out to 10^−9^. The dilution series was plated on trypticase soy agar with 5% bovine blood and incubated anaerobically for 6 days with plates being observed on days 2, 4, and 6. *Brachyspira* spp. grew confluently at the more concentrated dilutions, but discrete colonies were observed from the more dilute plates. Colonies were counted after 6 days incubation to obtain the inoculum titer in approximate colony-forming units per ml (CFU/ml). The approximate CFU/ml by day and isolate are presented in Table 2. Pigs in the control group received a sham inoculum consisting of agar material from non-inoculated culture plates prepared in the same manner as for the *Brachyspira* inocula.

**Table 2:** Approximate CFU/ml of *Brachyspira* spp. in inocula by days post inoculation and isolate.

Isolate	0 DPI* (CFU/ml)†	1 DPI (CFU/ml)	2 DPI (CFU/ml)
*Brachyspira hyodysenteriae* (B204)	1.4×10^6^	5.8×10^5^	8.8×10^5^
“*Brachyspira hampsonii*” (EB107)	1.1×10^6^	1.2×10^6^	1.2×10^6^
*Brachyspira pilosicoli* (BR2001)	8.9×10^7^	9.3×10^7^	7.9×10^7^
*Brachyspira intermedia* (BR2000)	9.0×10^5^	3.1×10^5^	8.8×10^5^

^*^DPI  =  days post inoculation.

† CFU/ml  =  colony-forming units per ml.

### Molecular identification

The subpassaged isolates used to prepare the inocula were verified to species by PCR amplification of partial *nox* gene sequences using previously described primers [12] followed by sequence comparison with sequences available in GenBank.

### Oral fluids

Oral fluids were collected from each group daily starting 5 DPI until two pigs remained within the group or at the termination of the study 21 DPI. Briefly, a 0.75 m length of 0.95 cm cotton rope was hung from a bracket in each pen for approximately 20 minutes. Following sufficient oral fluids accumulation, ropes were placed into a 3.8 L plastic bag and wrung by hand. The oral fluids were then transferred to a sterile plastic tube and submitted for isolation of *Brachyspira* spp. as previously described.

### Animal observations and necropsy

Throughout the study period, investigators were blinded to diet composition. Following inoculation, animals were observed at least twice daily for feed consumption, availability of adequate water, and clinical illness. Pigs were weighed at arrival, inoculation, and necropsy. Fecal consistency was determined daily, and each pig received a score based upon the following system: 0 if normal, 1 if soft but formed, 2 if semisolid, and 3 if liquid to watery with an additional 0.5 point added each for the presence of discernible mucus and/or blood. Animals were euthanized by barbiturate overdose within 72 hours of consecutive diarrhea with blood and mucus or at the termination of the study 21 DPI. At necropsy, the entire intestinal tract was observed for gross lesions and the full length of the cecal and colonic lumens were exposed and evaluated for the severity, distribution, and location of luminal mucus, mucosal hemorrhage, and fibrinous exudate. Tissue samples were collected and placed in 10% neutral buffered formalin and included sections of jejunum; ileum; cecum; base, apex, and a representative lesion from the spiral colon; descending colon; and liver. After 24 to 48 hours of fixation, tissue samples were transferred to 70% ethanol and the apex of the spiral colon and ileum were processed routinely for histopathology. A rectal swab was also collected from each pig for microbial culture. The pH of luminal contents taken from the apex and cecum was recorded using a calibrated commercially available portable pH meter (Thermo Scientific, Orion Star A121 pH Portable Meter) within 5 minutes of euthanasia.

### Histopathology

The investigator was blinded to individual pig numbers and group identifications. Sections were cut to 4 µm and stained routinely with hematoxylin and eosin. Sections of ileum were evaluated for the presence of any lesions suggestive of *Lawsonia intracellularis* or *Salmonella* spp. infection. For sections of spiral colon, neutrophils in the lamina propria were counted in ten 40X fields and the mean for each section of the apex was determined. Crypt depth was measured in each section using a standard eyepiece micrometer and measurements were taken from an area where the crypts were perpendicular to the mucosal surface with intact epithelium. The mean of three measurements was calculated for each section of apex of the spiral colon for each pig.

### Statistical analyses

Commercial statistical software packages (SAS 9.3, SAS Institute, Cary, NC; JMP Pro 10, SAS Institute, Cary, NC) were utilized to perform all analyses. Average daily gain (ADG) at 2 wks, total ADG, cecal pH, and pH at the apex of the spiral colon were analyzed using analyses of variance (ANOVA). Time to event data (days to SD, days to positive culture) were analyzed using Cox's proportional hazard regression. *Brachyspira* spp. inoculated, diet type, and their interaction were used as explanatory variables in the above analyses. Wilcoxon two-sample test was performed for difference in neutrophilic inflammation and crypt depth. In all circumstances, values of *P*≤0.05 were considered significant and means are reported with standard error of the mean.

## Results

### Animal observations

Clinical signs of dysentery were first observed 5 and 7 DPI in the *B. hyodysenteriae*-inoculated diet 2 group and *B. hyodysenteriae*-inoculated diet 1 group, respectively and 4 and 6 DPI in the “*B. hampsonii*”-inoculated diet 2 group and “*B. hampsonii*”-inoculated diet 1 group, respectively. The timing of onset of clinical dysentery for individual animals within each group is displayed in Tables 3 and 4. When analyzed based upon culture phenotype, the average DPI to the onset of clinical SD was significantly less in strongly beta-hemolytic-inoculated diet 2 groups than strongly beta-hemolytic-inoculated diet 1 groups (*P* = 0.018). However, when analyzed based upon inoculum identity and diet, the average DPI to the onset of clinical SD remained statistically significant only when comparing the *B. hyodysenteriae*-inoculated diet 2 group and *B. hyodysenteriae*-inoculated diet 1 group (diet 1: 12.2±1.5; diet 2: 6.8±0.33; *P* = 0.0009). For the “*B. hampsonii*”-inoculated groups the average DPI to the onset of SD was numerically shorter in the diet 2 group (diet 1: 12.4±1.45; diet 2: 8.0±0.46); however, this difference was not statistically significant. Fecal scores and timing of euthanasia for each pig are summarized by group in Table 3 and 4 for *B. hyodysenteriae* and “*B. hampsonii*” groups, respectively.

**Table 3:** Summary of fecal scores, *Brachyspira* culture results of feces and oral fluids, and timing of euthanasia of B204-inoculated pigs^a^^,^ b.

	Individual Fecal Scores^c^ by Days Post-Inoculation																
Diet Pig ID	5	6	7	8	9	10	11	12	13	14	15	16	17	18	19	20	21
*0% DDGS* d																	
88	0+	2+	4+	4+	4+	4+	-	-	-	-	-	-	-	-	-	-	-
83	0+	0+	4+	4+	4+	4+	-	-	-	-	-	-	-	-	-	-	-
85	0	0+	2+	4+	4+	4+	-	-	-	-	-	-	-	-	-	-	-
84	0+	0+	0+	0+	4+	4^+s^	4 +	-	-	-	-	-	-	-	-	-	-
86	0	0	0	1	0	0+	4+	4+	4+	4^+s^	-	-	-	-	-	-	-
87	0	0	0	0	0+	0+	1+	4+	4+	4+	-	-	-	-	-	-	-
81	0	1+	0	1	0	1	1+	1+	3.5+	4	2.5	2.5	1	1	1	0	1
82	0	1	0+	1	0+	0	0+	0	1	1+	0	0	0	0	0	0	0
89	0	0	0	0	0+	0	0	0	0	0	0	0+	0+	0	0	0	0
90	0	0	0+	0	0	0	0	0	0+	0+	0+	2.5+	1+	4+	4+	3+	4+
Oral Fluids	Neg	Neg	Neg	Pos	Pos	Pos	Pos	Pos	Pos	Pos	Neg	Pos	Neg	Pos	Pos	Neg	Pos
*30% DDGS*																	
92	3.5+	4+	4+	4 +	-	-	-	-	-	-	-	-	-	-	-	-	-
96	0+	4+	4+	4+	-	-	-	-	-	-	-	-	-	-	-	-	-
100	0+	4+	4+	4+	-	-	-	-	-	-	-	-	-	-	-	-	-
91	2+	3.5+	4+	4+	4^+s^	-	-	-	-	-	-	-	-	-	-	-	-
94	0+	4+	4+	4+	4+	-	-	-	-	-	-	-	-	-	-	-	-
95	0+	4+	4+	4+	4+	-	-	-	-	-	-	-	-	-	-	-	-
98	0+	0+	4+	4	4+	-	-	-	-	-	-	-	-	-	-	-	-
99	0	0+	4+	4+	4 +	-	-	-	-	-	-	-	-	-	-	-	-
93	0	0	0	0+	4+	4+	4+	-	-	-	-	-	-	-	-	-	-
97	0	0+	0+	4	4+	4+	4^+s^	-	-	-	-	-	-	-	-	-	-
Oral Fluids	Neg	Pos	Pos	Pos	Neg	-	-	-	-	-	-	-	-	-	-	-	-

a B204 = B. hyodysenteriae.

b Sham-inoculated diet 1 group and sham-inoculated diet 2 group had a maximum daily diarrhea score of 0.1 on days 11 and18 post-inoculation and 0.2 on day 14, respectively; never had a positive Brachyspira culture; and were euthanized at 21 days post inoculation.

c A fecal score was determined for each pig daily based upon the following system: 0 if normal, 1 if soft but formed, 2 if unformed with semisolid consistency, and 3 if severely liquid to watery with an additional 0.5 point added each for the presence of discernible mucus and/or blood (max score  = 4).

d Distillers dried grains with solubles.

+ Positive Brachyspira culture.

s Positive Salmonella culture at necropsy.

**Table 4:** Summary of fecal scores, Brachyspira culture results, and timing of euthanasia of EB107-inoculated pigs^a^^,^ b.

	Individual Fecal Scores^c^ by Days Post-Inoculation																
Diet Pig ID	5	6	7	8	9	10	11	12	13	14	15	16	17	18	19	20	21
*0% DDGS* d																	
70	3.5^+^	4^+^	4^+^	4^+^	-	-	-	-	-	-	-	-	-	-	-	-	-
69	2^+^	4^+^	4^+^	4^+^	4^+^	-	-	-	-	-	-	-	-	-	-	-	-
68	0	0^+^	1^+^	2.5^+^	4^+^	4^+^	4^+s^	-	-	-	-	-	-	-	-	-	-
62	0	0	0^+^	0^+^	0^+^	1^+^	4^+^	4^+^	4^+^	4^+^	-	-	-	-	-	-	-
63	1	1^+^	1	1^+^	0^+^	1^+^	2.5^+^	4^+^	4^+^	4^+^	-	-	-	-	-	-	-
67	0	0	0^+^	0^+^	0^+^	0^+^	0^+^	4^+^	4^+^	4^+^	-	-	-	-	-	-	-
61	0	0^+^	0^+^	0	0^+^	0^+^	0	0	0	0	0	0	0	0	1	0^+^	1^+^
64	0	0	0	0^+^	0^+^	1^+^	0^+^	0	0^+^	0	0	0	4^+^	4^+^	3^+^	4^+^	4^+^
65	0^+^	0	0	0	0	0^+^	0	0^+^	0^+^	0	0	0	0	0	0^+^	0^+^	0^+^
66	0	0	0	0	0	0	0	0	0	0	0	0	0	0	0	0^+^	0^+^
Oral fluids	Pos	Pos	Pos	Pos	Pos	Pos	Pos	Pos	Pos	Pos	Neg	Neg	Pos	Pos	Pos	Pos	Pos
*30% DDGS*																	
76	4^+^	4^+^	-	-	-	-	-	-	-	-	-	-	-	-	-	-	-
79	0^+^	4^+^	4^+^	4^+s^	-	-	-	-	-	-	-	-	-	-	-	-	-
78	0^+^	0^+^	3.5^+^	4^+^	4^+^	4^+^	-	-	-	-	-	-	-	-	-	-	-
75	0	0	1^+^	4^+^	4^+^	4^+^	4^+^	-	-	-	-	-	-	-	-	-	-
77	0^+^	0^+^	1^+^	4^+^	4^+^	4^+^	4^+^	-	-	-	-	-	-	-	-	-	-
80	0^+^	0	0^+^	1^+^	4^+^	4^+^	4^+^	-	-	-	-	-	-	-	-	-	-
71	0	0	0	0	0	0^+^	0	0	0	0	0	0	0	0	0	0	0
72	0	0	0	0^+^	0	0^+^	0	0	0	0	0	0	0	0	0	0	0
73	0	0	0^+^	0^+^	0	0	0	0	0	0	0	0	0	0	0	0	0^+^
74	0	0	0^+^	0^+^	0^+^	0	0	0	0	0	0	0	0	0	0	0^+^	0
Oral fluids	Neg	Neg	Pos	Neg	Pos	Pos	Pos	Neg	Neg	Neg	Neg	Neg	Neg	Neg	Neg	Neg	Neg

a EB107  =  “*B. hampsonii*” clade II.

b Sham-inoculated diet 1 group and sham-inoculated diet 2 group had a maximum daily diarrhea score of 0.1 on days 11 and18 post-inoculation and 0.2 on day 14, respectively; never had a positive *Brachyspira* culture; and were euthanized at 21 days post inoculation.

c A fecal score was determined for each pig daily based upon the following system: 0 if normal, 1 if soft but formed, 2 if unformed with semisolid consistency, and 3 if severely liquid to watery with an additional 0.5 point added each for the presence of discernible mucus and/or blood (max score  = 4).

d Distillers dried grains with solubles.

+Positive *Brachyspira* culture.

s Positive *Salmonella* culture at necropsy.

Diarrhea with mild mucus was first noted 6 DPI in both *B*. *pilosicoli-*inoculated groups. The average maximum daily diarrhea score for pigs in the *B*. *pilosicoli-*inoculated diet 1 and *B*. *pilosicoli-*inoculated diet 2 groups was 0.85 on DPI 10 and 1.75 on DPI 10, respectively. No significant diarrhea was noted in either group of *B. intermedia*-inoculated pigs throughout the study period with a maximum average daily diarrhea score of 0.3 on DPI 5 for diet group 1 and 0.2 on DPI 9 for diet group 2. Similarly, sham-inoculated pigs had a maximum average daily diarrhea score of 0.1 on DPI 11 and 18 for diet group 1 and 0.2 on DPI 14 for diet group 2.

### Microbial culture

Oral fluids were commonly culture positive when collected from pens containing pigs shedding strongly beta-hemolytic spirochetes, and these results are presented by group in Table 3 and 4. Oral fluids collected from *B. pilosicoli*-inoculated pigs were positive 10 days out of the 17 days in which there was at least one culture positive rectal swab in diet group 1 and 8 days out of the 17 days in diet group 2 (daily culture data not shown). Oral fluids collected from *B. intermedia*-inoculated pigs were positive 8 days out of the 17 days in which there was at least one culture positive rectal swab in diet group 1 and 1 day out of the 16 days in diet group 2 (daily culture data not shown). Oral fluids collected from sham-inoculated pens were culture negative throughout the study.

Culture results for each pig are summarized by inoculation group and diet in Table 3 and 4 for *B. hyodysenteriae* and “*B. hampsonii*” groups, respectively. Days to first positive culture was shorter in both *B. hyodysenteriae*-inoculated diet 2 (mean ± SEM  = 5.5±0.30) and “*B. hampsonii*”-inoculated diet 2 groups (6.4±0.54) when compared to *B. hyodysenteriae*-inoculated diet 1 (6.9±0.59) and “*B. hampsonii*”-inoculated diet 1 (7.5±1.42) groups, respectively; however, this difference was only statistically significant when comparing the *B. hyodysenteriae*-inoculated groups (*P* = 0.0443). In contrast, the average days to first positive culture was longer in both *B. pilosicoli*-inoculated diet 2 (6.2±0.51) and *B. intermedia*-inoculated diet 2 (10.2±1.62) groups when compared to *B. pilosicoli*-inoculated diet 1 (5.3±0.15) and *B. intermedia*-inoculated diet 1 (8.56±1.09) groups, respectively; however, these differences were not statistically significant. Every pig in the *B. pilosicoli*-inoculated groups shed at least eight days throughout the study period while only 9 and 5 pigs shed viable spirochetes in the *B. intermedia*-inoculated diet 1 and 2 group, respectively. Sham-inoculated pigs were culture negative throughout the study.

At necropsy *Salmonella* spp. were isolated from 12 out of 100 pigs with the majority of these isolates (8 of 12) occurring only after tetrathionate broth enrichment. Pigs with positive cultures were distributed across multiple groups as follows: sham-inoculated diet 1 (1 of 10), sham-inoculated diet 2 (0 of 10), *B. pilosicoli* diet 1 (2 of 10), *B. pilosicoli* diet 2 (0 of 10), *B. intermedia* diet 1 (3 of 10), *B. intermedia* diet 2 (0 of 10), *“B. hampsonii”* diet 1 (1 of 10), *“B. hampsonii”* diet 2 (1 of 10), *B. hyodysenteriae* diet 1 (2 of 10), and *B. hyodysenteriae* diet 2 (2 of 10). Isolates were characterized as either serogroup B (10 of 12) or rarely C1 (2 of 12). Individual pigs with positive *Salmonella* culture are identified in Tables 3 and 4.

### Average daily gain

Average daily gain was not statistically different between any groups following the two weeks of diet acclimation. Average daily gain, as calculated from study initiation to study termination or euthanasia, was significantly less in pigs inoculated with strongly beta-hemolytic *Brachyspira* spp. than sham-inoculated or weakly beta-hemolytic-inoculated pigs (*P*<0.0001, all analyses). However, no statistically significant difference was identified between sham-inoculated or weakly beta-hemolytic-inoculated pigs (*P*>0.9, all analyses). Average daily gain is summarized by group in Table 5.

**Table 5:** Average daily gain by group.

Group	ADG^a^	SEM
Sham-inoculated diet 1^b^	0.90	0.05
Sham-inoculated diet 2^c^	0.85	0.04
*B. intermedia*-inoculated diet 1	0.90	0.04
*B. intermedia*-inoculated diet 2	0.90	0.04
*B. pilosicoli*-inoculated diet 1	0.84	0.03
*B. pilosicoli*-inoculated diet 2	0.90	0.03
“*B. hampsonii*”-inoculated diet 1	0.60*	0.09
“*B. hampsonii*”-inoculated diet 2	0.62*	0.09
*B. hyodysenteriae*-inoculated diet 1	0.57*	0.06
*B. hyodysenteriae*-inoculated diet 2	0.47*	0.04

a Mean of Average Daily Gain (kg/day).

b Diet with 0% DDGS.

c Diet with 30% DDGS.

^*^Significantly different from the sham-inoculated and weakly beta-hemolytic inoculated group regardless of diet (*P*<0.0001, all analyses).

### Large intestine pH

Cecal pH was more alkaline in the sham-inoculated diet 2 group (6.157±0.06) than the sham-inoculated diet 1 group (5.906±0.05), and this difference was statistically significant (*P* = 0.0025). No statistically significant difference was observed within any other group between diet 1 and diet 2 (*P*>0.17, all analyses). The pH at the apex was significantly different between sham-inoculated diet 1 group (6.169±0.05) and the sham-inoculated diet 2 group (6.389±0.07; *P* = 0.01).

### Gross pathology

Gross lesions observed in the *B. hyodysenteriae* and “*B. hampsonii*” groups are summarized in Table 6. When present, gross lesions in these groups were limited to the cecum and large intestine. Lesions consisted of variable degrees of fibrinous exudate; mucosal thickening, congestion, and hemorrhage; and luminal mucus. No gross lesions were observed in the sham-inoculated groups, *B. intermedia*-inoculated groups, or *B. pilosicoli*-inoculated diet 1 group. Two pigs in the *B. pilosicoli*-inoculated diet 2 group had mild multifocal mucofibrinous exudate present in the centripetal portion and apex of the spiral colon.

**Table 6:** Summary of gross lesions of “*B. hampsonii*”-inoculated and *B. hyodysenteriae*-inoculated groups.

	Mucosal Fibrinous Exudate										
	Severity		Distribution			Location					
Group	Mild	M/S	MF	Seg	D	Cecum	Base	CP	Apex	CF	DC
“*B. hampsonii*”-inoculated diet 1^a^	6/10	1/10	4/10	3/10	0/10	5/10	4/10	5/10	2/10	1/10	0/10
“*B. hampsonii*”-inoculated diet 2^b^	4/10	0/10	2/10	2/10	0/10	2/10	2/10	3/10	3/10	1/10	0/10
*B. hyodysenteriae*-inoculated diet 1	3/10	4/10	4/10	1/10	1/10	5/10	6/10	7/10	6/10	3/10	3/10
*B. hyodysenteriae*-inoculated diet 2	1/10	9/10	1/10	5/10	4/10	9/10	10/10	9/10	7/10	2/10	1/10

	Luminal Mucus										
	Severity		Distribution			Location					
	Mild	M/S	MF	Seg	D	Cecum	Base	CP	Apex	CF	DC
“*B. hampsonii*”-inoculated diet 1	1/10	8/10	0/10	2/10	7/10	2/10	7/10	9/10	8/10	7/10	7/10
“*B. hampsonii*”-inoculated diet 2	2/10	5/10	1/10	1/10	5/10	4/10	5/10	7/10	6/10	6/10	4/10
*B. hyodysenteriae*-inoculated diet 1	2/10	5/10	0/10	0/10	7/10	4/10	7/10	7/10	7/10	7/10	5/10
*B. hyodysenteriae*-inoculated diet 2	0/10	10/10	0/10	0/10	10/10	6/10	9/10	10/10	10/10	10/10	9/10

	Hemorrhage										
	Severity		Distribution			Location					
	Mild	M/S	MF	Seg	D	Cecum	Base	CP	Apex	CF	DC
“*B. hampsonii*”-inoculated diet 1	0/10	7/10	5/10	0/10	2/10	2/10	5/10	7/10	7/10	6/10	6/10
“*B. hampsonii*”-inoculated diet 2	4/10	3/10	5/10	2/10	0/10	4/10	4/10	6/10	6/10	6/10	4/10
*B. hyodysenteriae*-inoculated diet 1	4/10	3/10	5/10	1/10	1/10	3/10	4/10	6/10	7/10	4/10	3/10
*B. hyodysenteriae*-inoculated diet 2	3/10	7/10	10/10	0/10	0/10	5/10	9/10	10/10	10/10	9/10	8/10

M/S  =  Moderate or severe.

MF  =  Multifocal.

Seg  =  Segmental.

D =  Diffuse.

CP  =  Centripetal.

CF  =  Centrifugal.

DC  =  Descending colon.

a Diet with 0% DDGS.

b Diet with 30% DDGS.

### Histopathology

Histologic evaluation of the apex of the spiral colon is summarized in Table 7. At the apex of the spiral colon there was a significant increase in neutrophilic inflammation when comparing each strongly beta-hemolytic-inoculated group and each sham-inoculated group or each weakly beta-hemolytic-inoculated group regardless of diet (*P*<0.02, all analyses). There was no significant difference in neutrophilic inflammation between strongly beta-hemolytic-inoculated groups (*P*>0.18, all analyses). Crypt depth was statistically different when comparing the *B. hyodysenteriae*-inoculated groups and the “*B. hampsonii*”-inoculated diet 1 group to the sham-inoculated groups as well as each weakly beta-hemolytic-inoculated groups (*P*<0.02, all analyses). There was no significant difference in crypt depth between strongly beta-hemolytic-inoculated groups or weakly beta-hemolytic-inoculated groups and sham-inoculated control groups.

**Table 7:** Summary of histologic lesions in the apex of the spiral colon.

	Neutrophilic Inflammation^a^		Crypt Depth (µm)^b^	
Group	Mean	SEM	Mean	SEM
Sham-inoculated diet 1 c	0.58	0.17	466.70	12.47
Sham-inoculated diet 2^d^	1.10	1.17	506.70	24.78
*B. intermedia*-inoculated diet 1	0.46	0.24	478.90	18.52
*B. intermedia*-inoculated diet 2	0.44	0.18	469.70	12.95
*B. pilosicoli*-inoculated diet 1	0.12	0.03	496.50	14.34
*B. pilosicoli*-inoculated diet 2	0.34	0.11	518.00	21.29
“*B. hampsonii*”-inoculated diet 1	29.38*	5.44	848.90*	57.38
“*B. hampsonii*”-inoculated diet 2	19.34*	6.45	696.70	79.60
*B. hyodysenteriae*-inoculated diet 1	24.85*	7.62	763.90*	64.78
*B. hyodysenteriae*-inoculated diet 2	25.17*	3.74	799.00*	32.33

a Neutrophils were counted in ten 40X fields and the mean for each section of the spiral colon was determined.

b Crypt depth was determined by taking the mean of three measurements for each section of apex of the spiral colon.

c Diet with 0% DDGS.

d Diet with 30% DDGS.

^*^Significantly different from the sham-inoculated and weakly beta-hemolytic inoculated group regardless of diet (*P*<0.02, all analyses).

## Discussion

A resurgence of swine dysentery has recently been observed in North American swine herds after a period in which the disease was nearly eliminated. The underlying reason(s) for this increased observance is poorly understood; however, coincident with the reemergence of *Brachyspira*-associated disease in pigs has been increased feeding of insoluble dietary fiber through the addition of DDGS in many swine diets.

Currently, relatively little is published about the precise pathogenesis of SD; however, diet is considered to play a major role in disease expression [7]. The precise mechanisms by which diet composition impacts the pathophysiology of SD continue to be elusive. In a previous study, pigs fed a highly digestible diet of cooked rice developed an increased colonic content pH and decreased total volatile fatty acid concentrations and were protected from developing SD [8]. The protective mechanism of such a diet could be due to decreased fermentation in the large intestine [8] and/or changes in the microbiota with a subsequent increase in species that inhibit colonization of *B. hyodysenteriae* [13], [14], [15]. Addition of rapidly fermentable fiber, which produce the opposite effect than highly digestible diets in the hindgut, to the highly digestible diet of cooked rice resulted in reinstatement of susceptibility to swine dysentery [16]. The protective affect of rice was not corroborated by a later study [17] where parboiled rice again alkalinized colonic contents yet failed to protect pigs from infection with *B. hyodysenteriae* and development of SD suggesting that perhaps the type of rice may be important. Although a highly digestible diet has been associated with protection against SD, this has not always been the case illustrating the multifactorial and complex nature of the pathophysiology of SD [18], [19].

Another study using rapidly fermentable carbohydrates [20] protected against SD resulting in further discord in the literature. Yet, the diet was based on dried chicory roots and sweet lupins leading to speculation that the protective effect was due to the presence of inulin in dried chicory roots [21] as a diet rich in inulin has been shown to prevent colonization of *B*. *hyodysenteriae* [21], [22]. This protective mechanism is again not well characterized. The inclusion of inulin in swine diets decreases the protein: carbohydrate ratio in the hindgut increasing the lactate and butyrate-producing bacteria [15], [23] and decreasing proteolytic bacteria. Proteolytic bacteria may be synergistic with *B*. *hyodysenteriae* in SD pathogenesis especially in the breakdown of glycoproteins which are a major component of the protective mucus bilayer. In the end, it appears that the ingredient digestibility plays a major role in the pathophysiology of disease through the manipulation of the large intestinal microbiota [24], [25].

The inclusion of 30% DDGS in the ration of this study reflects a practical level, relative to industry practice, while adding sufficient insoluble dietary fiber to adequately test the hypothesis [10]. A recent study found that feeding 30% DDGS did not affect the concentration of volatile fatty acids in ileal, cecal, or fecal samples; however, the pH of ileal and cecal digesta from pigs fed the DDGS diet was greater than pigs fed the control diet [26]. Similarly in the current study, the pH at the cecum and apex was significantly more alkaline in the sham-inoculated diet 2 group (30% DDGS) than the sham-inoculated diet 1 group (0% DDGS). Yet, this alkalization of the colonic contents was not associated with protection against the development of SD as previously seen in other studies as not only did pigs in the diet 2 groups inoculated with strongly beta-hemolytic spirochetes develop SD but the average days post inoculation to the onset of clinical SD was significantly shorter. Furthermore, the days to first positive culture was also significantly shorter when comparing the *B. hyodysenteriae*-inoculated diet 2 group and *B. hyodysenteriae*-inoculated diet 1 group. Such incongruence concerning the impact of colonic content pH could be attributed to the multifactorial nature of SD in which the pH of the large intestinal content is a single component amongst many that influence the clinical expression of SD including fermentation in the large intestine, mucolytic enzyme activity, and/or the total microbiota.

The large intestine contains a dynamic microenvironment with tremendous interplay between microorganisms. The enteric microbiome can change rapidly in a diet-specific manner [27] resulting in changes in goblet cell functions and in the composition of intestinal mucus through the production of bioactive factors by epithelial and lamina proprial inflammatory cells or direct stimulation of signaling cascades. Dietary fiber has been shown to increase viscosity and production of mucins [28]. Specifically, insoluble fiber increases daily excretion of fucose, galactose, and mucins [29] all of which are chemoattractant to *B. hyodysenteriae* [30] and the last of which is chemoattractant to *B. pilosicoli* [31], suggesting a link between increased mucus production and the propensity for colonization by *Brachyspira* spp. In the current study, increased insoluble dietary fiber through the feeding of DDGS shortened the time to onset of dysentery in pigs inoculated with strongly beta-hemolytic *Brachyspira* spp. The observed effect was most significant between the *B. hyodysenteriae*-inoculated diet groups, but was also apparent in the “*B. hampsonii*”-inoculated groups and the lack of statistical significance with this species may reflect the total number of pigs that developed disease and limitations of statistical analysis on time to event data rather than an actual difference in disease pathophysiology. The feeding of increased insoluble dietary fiber did not have any significant observable effect on pigs inoculated with either *B. pilosicoli* or *B. intermedia* under the conditions of the current study. A potential limitation of this study is the differential impact, if any, of standard media on the growth cycle of the different *Brachyspira* spp. While log phase growth can be determined using broth culture, this determination cannot be made using an agar slurry inoculum as used in this study.

While a few pigs had positive *Salmonella* cultures at necropsy, most of these cultures were from tetrathionate broth enrichment of samples set up within a few hours of collection. This low level of recovery and requirement for enrichment suggests these were likely subclinically infected pigs and this is further supported by the lack of lesions typical of salmonellosis in these pigs. Furthermore, the *Salmonella* positive pigs were fairly evenly distributed across inoculated groups which should further minimize any potential confounding effects of this minor coinfection on the parameters assessed.

Recent analysis of the *B. hyodysenteriae* genome has revealed multiple metabolic pathways and enzymes that could be utilized as a competitive advantage with the addition of dietary insoluble fiber such as DDGS [32]. Fermentation of fiber produces short-chain fatty acids [33] which *B. hyodysenteriae* has the capacity to use as a major energy and carbon source [32]. Additionally, *B. hyodysenteriae* has many amino acid and oligopeptide transporters that may enhance the utilization of amino acids as a major energy source [32]. Furthermore, *B. hyodysenteriae* possesses key enzymes for gluconeogenesis as well as fifteen proteases, all of which suggests that a shift to protein fermentation in the colon may provide multiple potential energy sources for the pathogenic *Brachyspira* sp. [32]. Future genetic analyses of the genome of “*B. hampsonii*” are needed to determine if similar or distinctly different pathways and enzymes are present which may account for the observations in the present study.

Diet composition alone had no significant impact on ADG over the time course of the present study when comparing the sham-inoculated groups. A highly significant difference in ADG was observed between *B. hyodysenteriae*-inoculated or “*B. hampsonii*”-inoculated groups compared with sham-inoculated or weakly-beta hemolytic-inoculated groups regardless of diet. This highly significant reduction in ADG is consistent with what is reported historically with SD and underscores the ongoing significance of this pathogen in worldwide pig production. No significant difference was observed when comparing the *B. pilosicoli*-inoculated or *B. intermedia*-inoculated groups to the sham-inoculated groups under the conditions of this study. This was unexpected given the historical association of *B. pilosicoli* with PCS and the high percentage of pigs that became culture positive and shed the organism for a prolonged period. These findings would suggest that the economic impact of these spirochetes may result from infections lasting longer than the conditions of this report or from infections coincident with other factors.

In swine, oral fluids can be used to detect both viral and bacterial pathogens including porcine reproductive and respiratory syndrome virus, swine influenza virus, and *Mycoplasma hyopneumoniae* by PCR. Oral fluids are easily acquired and represent a pen level sample allowing for detection of pathogens across multiple pigs. Results of this investigation support the use of oral fluids as an additional sample type for the detection of *Brachyspira* spp. by culture in pigs with clinical disease.

In the present study, there were minimal differences in lesion characteristics, severity, or distribution between pigs infected with either *B*. *hyodysenteriae* or “*B*. *hampsonii*” clade II. These findings are consistent with previous reports [4]–[6] and suggest that while feeding DDGS may increase the incidence of SD it may not necessarily impact the severity of disease observed.

In summary, the results of the present study reveal that pigs receiving 30% DDGS shed on average one day prior to and developed swine dysentery nearly twice as fast as pigs receiving 0% DDGS. Accordingly, dietary composition should be considered an important risk factor for the development of SD and dietary modification might also be an additional consideration in any effective disease elimination strategy for strongly beta-hemolytic *Brachyspira* spp. infections.

## References

[1] Harris DL, Glock RD, Christensen CR, Kinyon JM (1972) Inoculation of pigs with *Treponema hyodysenteriae* (new species) and reproduction of the disease. Vet Med Small Anim Clin 67:61–64. 4480857

[2] Trott DJ, Stanton TB, Jensen NS, Duhamel GE, Johnson JL, et al (1996) *Serpulina pilosicoli* sp. nov., the agent of porcine intestinal spirochetosis. Int J Syst Bacteriol 46:206–215. 8573497 10.1099/00207713-46-1-206

[3] Chander Y, Primus A, Oliveira S, Gebhart CJ (2012) Phenotypic and molecular characterization of a novel strongly hemolytic *Brachyspira* species, provisionally designated "*Brachyspira hampsonii*". J Vet Diagn Invest 24:903–910. 22914820 10.1177/1040638712456975

[4] Burrough ER, Strait EL, Kinyon JM, Bower LP, Madson DM, et al (2012) Comparative virulence of clinical *Brachyspira* spp. isolates in inoculated pigs. J Vet Diagn Invest 24:1025–1034. 22956484 10.1177/1040638712457927

[5] Rubin JE, Costa MO, Hill JE, Kittrell HE, Fernando C, et al (2013) Reproduction of mucohaemorrhagic diarrhea and colitis indistinguishable from swine dysentery following experimental inoculation with "*Brachyspira hampsonii*" strain 30446. PLoS One 8:e57146. 23460829 10.1371/journal.pone.0057146 PMC3584117

[6] Wilberts BL, Arruda PH, Kinyon JM, Madson DM, Frana TS, et al (2014) Comparison of lesion severity, distribution, and colonic mucin expression in pigs with acute swine dysentery following oral inoculation with "*Brachyspira hampsonii*" or *Brachyspira hyodysenteriae* . Vet Pathol 51:1096–1108. 24577722 10.1177/0300985813516646

[7] Hampson DJ (2012) Brachyspiral colitis. In:Zimmerman JJ, Karriker LA, Ramirez A, Schwartz KJ, Stevenson GWeditors. Diseases of swine.Ames, IA: Wiley-Blackwell. pp.680–696.

[8] Siba PM, Pethick DW, Hampson DJ (1996) Pigs experimentally infected with *Serpulina hyodysenteriae* can be protected from developing swine dysentery by feeding them a highly digestible diet. Epidemiol Infect 116:207–216. 8620913 10.1017/s0950268800052456 PMC2271630

[9] NRC (2012) Nutrient Requirements of Swine. Washington, D.C.: The National Academies Press.

[10] Urriola PE, Shurson GC, Stein HH (2010) Digestiblity of dietary fiber in distillers co-products fed to growing pigs. J Anim Sci 88:2373–2381. 20228242 10.2527/jas.2009-2227

[11] Warneke HL, Kinyon JM, Bower LP, Burrough ER, Frana TS (2014) Matrix-assisted laser desorption ionization time-of-flight mass spectrometry for rapid identification of *Brachyspira* species isolated from swine, including the newly described "*Brachyspira hampsonii*.". J Vet Diagn Invest 26:635–639. 25012082 10.1177/1040638714541114

[12] Rohde J, Rothkamp A, Gerlach GF (2002) Differentiation of porcine *Brachyspira* species by a novel *nox* PCR-based restriction fragment length polymorphism analysis. J Clin Microbio 4:2598–2600. 10.1128/JCM.40.7.2598-2600.2002 PMC120595 12089283

[13] Klose V, Bruckbeck R, Henikl S, Schatzmayr G, Loibner AP (2010) Identification and antimicrobial susceptibility of porcine bacteria that inhibit the growth of *Brachyspira hyodysenteriae* in vitro. J Appl Microbiol 108:1271–1280. 19778354 10.1111/j.1365-2672.2009.04521.x

[14] Leser TD, Lindecrona RH, Jensen TK, Jensen BB, Møller K (2000) Changes in bacterial community structure in the colon of pigs fed different experimental diets and after infection with *Brachyspira hyodysenteriae* . Appl Environ Microbiol. 66:3290–3296. 10919783 10.1128/aem.66.8.3290-3296.2000 PMC92147

[15] Mølbak L, Thomsen LE, Jensen TK, Back Knudsen KE, Boye M (2007) Increased amount of *Bifidobacterium thermacidophilum* and *Megasphaera elsdenii* in the colonic microbiota of pigs fed a swine dysentery preventive diet containing chicory roots and sweet lupine. J Appl Microbiol 103:1853–67. 17953596 10.1111/j.1365-2672.2007.03430.x

[16] Pluske JR, Durmic Z, Pethick DW, Mullan BP, Hampson DJ (1998) Confirmation of the role of rapidly fermentable carbohydrates in the expression of swine dysentery in pigs after experimental infection. J Nutr 128:1737–44. 9772144 10.1093/jn/128.10.1737

[17] Kirkwood RN, Huang SX, McFall M, Aherne FX (2000) Dietary factors do not influence the clinical expression of swine dysentery. Swine Health Prod 8:73–76.

[18] Durmic Z, Pethick DW, Mullan BP, Schulze H, Accioly JM, et al (2000) Extrusion of wheat or sorghum and/or addition of exogenous enzymes to pig diets influences the large intestinal microbiota but does not prevent development of swine dysentery following experimental challenge. J Appl Microbiol 89:678–686. 11054173 10.1046/j.1365-2672.2000.01166.x

[19] Lindecrona RH, Jensen TK, Jensen BB, Leser TD, Jiufeng W, et al (2003) The influence of diet on the development of swine dysentery upon experimental infection. Anim Sci 76:81–87.

[20] Thomsen LE, Bach Knudsen KE, Jensen TK, Christensen AS, Møller K, et al (2007) The effect of fermentable carbohydrates on experimental swine dysentery and whip worm infections in pigs. Vet Microbiol 119:152–163. 17049759 10.1016/j.vetmic.2006.09.004

[21] Hansen CF, Phillips ND, La T, Hernandez A, Mansfield J, et al (2010) Diets containing inulin but not lupins help to prevent swine dysentery in experimentally challenged pigs. J Anim Sci 88:3327–3336. 20525927 10.2527/jas.2009-2719

[22] Hansen C, Hernández A, Mansfield J, Hidalgo Á, La T, et al (2011) A high dietary concentration of inulin is necessary to reduce the incidence of swine dysentery in pigs experimentally challenged with *Brachyspira hyodysenteriae* . Br J Nutr 106:1506–1513. 21736788 10.1017/S000711451100208X

[23] Cummings JH, Macfarlane GT (1991) The control and consequences of bacterial fermentation in the human colon. *J Appl Bacteriol* 70:443–459. 1938669 10.1111/j.1365-2672.1991.tb02739.x

[24] Dréau D, Lallés JP, Philouze-Romé V, Toullec R, Salmon H (1994) Local and systemic immune responses to soybean protein ingestion in early-weaned pigs. J Anim Sci 72:2090–2098. 7982839 10.2527/1994.7282090x

[25] Huisman J, Jansman AJM (1991) Dietary effects and some analytical aspects of antinutritional factors in pea (*Pisum sativum*), common beans (*Phaseolus vulgaris*) and soyabeans (*Glycine max* L.) in monogastric farm animals. A literature review. Nutr Abstr Rev 61:901–921.

[26] Urriola PE, Stein HH (2010) Effects of distillers dried grains with solubles on amino acid, energy, and fiber digestibility and on hindgut fermentation of dietary fiber in a corn-soybean meal diet fed to growing pigs. J Anim Sci 88:1454–1462. 20023135 10.2527/jas.2009-2162

[27] David LA, Maurice CF, Carmody RN, Gootenberg DB, Button JE, et al (2014) Diet rapidly and reproducibly alters the human gut microbiome. Nature 505:559–563. 24336217 10.1038/nature12820 PMC3957428

[28] Schmidt-Wittig U, Enss ML, Coenen M, Gärtner K, Hedrich HJ (1996) Response of rat colonic mucosa to a high fiber diet. Ann Nutr Metab 40:343–350. 9087313 10.1159/000177936

[29] Montagne L, Piel C, Lalles JP (2004) Effect of diet on mucin kinetics and composition: nutrition and health implications. Nutr Rev 62:105–114. 15098857 10.1111/j.1753-4887.2004.tb00031.x

[30] Kennedy MJ, Yancey RJ Jr (1996) Motility and chemotaxis in *Serpulina hyodysenteriae* . Vet Microbiol 49:21–30. 8861640 10.1016/0378-1135(95)00174-3

[31] Naresh R, Hampson DJ (2010) Attraction of *Brachyspira pilosicoli* to mucin. Microbiology 156:191–197. 19833772 10.1099/mic.0.030262-0

[32] Bellgard MI, Wanchanthuek P, La T, Ryan K, Moolhuijzen P, et al (2009) Genome sequence of the pathogenic intestinal spirochete *Brachyspira hyodysenteriae* reveals adaptations to its lifestyle in the porcine large intestine. PLoS One 4:e4641. 19262690 10.1371/journal.pone.0004641 PMC2650404

[33] Zijlstra RT, Jha R, Woodward AD, Fouhse J, van Kempen TA (2012) Starch and fiber properties affect their kinetics of digestion and thereby digestive physiology in pigs. J Anim Sci 90:49–58. 23365281 10.2527/jas.53718

